# What You Need to Know About the Ebola Virus

**Published:** 2014-11-01

**Authors:** Wendy H. Vogel, Pamela Hallquist Viale

Although the *Journal of the Advanced Practitioner in Oncology* (JADPRO) is focused on publishing issues and content specific to oncology, we recognize that the current outbreak of the Ebola virus is a critical issue and one that readers should be aware of.

The Ebola virus was first noted in 1976, when two outbreaks occurred in the Sudan and the Democratic Republic of the Congo. The adjacent Ebola River in the Congo location is where the hemorrhagic fever gets its name. Periodic outbreaks in Africa have occurred since its discovery (World Health Organization [WHO], 2014). The current outbreak is the longest of all known outbreaks—and the deadliest—with ~5,000 deaths as of this month (Centers for Disease Control and Prevention [CDC], 2014). As we go to press, the CDC reports 8,168 laboratory-confirmed cases as well.

The Ebola virus affects both humans and nonhuman primates and is spread by close contact with patients or exposure to infected biologic fluids. The virus has been found in the blood, saliva, feces, breast milk, tears, and genital secretions of infected patients (Chippaux, 2014).

The Ebola virus has a high mortality rate, with a current case fatality rate estimated to be around 50%. Mortality rates for previous outbreaks have varied from 25% to 90%, and those outbreaks have been relatively short-lived (WHO, 2014). The onset of symptoms for infection with the Ebola virus varies, given the fact that the incubation period is 2 to 21 days (WHO, 2014). Infection with the Ebola virus causes significant immune suppression and a systemic inflammatory response, leading to multiorgan failure and shock (Gajdosik, 2014). Patients may develop vomiting and diarrhea; kidney and liver function may be affected; and some patients may experience bleeding, both internal and external (WHO, 2014).

Treatment for infection with the virus is largely supportive care, as rehydration and treatment of symptoms improve survival in affected patients. Although experimental vaccines are in the testing phase, adequate control of the outbreak is essential, requiring management of cases, surveillance, and contact tracing (WHO, 2014). Wearing appropriate protective gear while caring for this population of patients is critical to prevention of virus transmission.

On the next pages you’ll find several resources for health-care professionals interested in the most current and relevant information on the Ebola virus. Revisit these sites frequently, as information regarding the virus and its treatments as well as guidelines for prevention of transmission are constantly updated.

## Online Resources

**CDC Interim US Guidance for Monitoring and Movement of Persons With Potential Ebola Virus Exposure**

http://www.cdc.gov/vhf/ebola/pdf/monitoring-and-movement.pdf

Updated guideline on the monitoring and movement of persons potentially infected with the Ebola virus to reduce the risk of transmission of the virus

**Ebola: Donning and Doffing of Personal Protective Equipment**

http://www.medscape.com/viewarticle/833907?src=wnl_edit_specol&uac=45381CJ

Includes a video showing step-by-step information regarding the donning of protective equipment to prevent transmission of disease

**Oxford University Press Resources for Ebola World Health Emergency**

http://www.oxfordjournals.org/en/our-journals/medicine-and-health/ebola.html?gclid=CjwKEAjww8eiBRCE7qvK9Z7W_DgSJABfOjf2kzpJJgLpj1rRK0pvliTccNypHU5vVkeUplIwnnvmhRoCfJTw_wcB

Free access (until January 4, 2016) to more than 50 articles from leading journals and online resources freely accessible to assist researchers, medical professionals, policy makers, and others working on the containment, treatment, and prevention of Ebola virus disease

**Centers for Disease Control and Prevention Communication Resources**

http://www.cdc.gov/vhf/ebola/resources/index.html?s_cid=cs_021

Audio, video, infographics, fact sheets, banners, posters, and brochures; useful links and information for travelers and health-care workers

**World Health Organization Global Alert and Response**

http://www.who.int/csr/resources/publications/ebola/en/

Technical information, information on preparedness and response, infection control, patient care, laboratory collection safety, epidemiology, social mobilization, clinical management, and publications by WHO; PDFs on how to put on and remove personal protective equipment; checklist on Ebola virus disease preparedness

**The New England Journal of Medicine**

http://www.nejm.org/page/ebola-outbreak

Collection of articles and other resources on the Ebola outbreak, including clinical reports, management guidelines, and commentary

**The Lancet Ebola Resource Centre**

http://ebola.thelancet.com

Research, reviews, editorials, correspondence, world report, archive, and resources

**American Medical Association Ebola Resource Center**

http://www.ama-assn.org/ama/pub/physician-resources/public-health/ebola-resource-center.page

Resources for public and health-care providers, preparing hospitals/practice, screening/diagnosing Ebola, treatment, travel and infection, news and other resources

**US Department of Health & Human Services, Disaster Information Management Research Center**

http://sis.nlm.nih.gov/dimrc/ebola_2014.html

Information resources, US federal organizations, international organizations, international governments, and free resources from various publishers; biomedical journal literature and resources, training resources, outbreak map, public resources, multilanguage resources, clinical trials, and situation reports; includes Twitter 

**American Hospital Association Ebola Preparedness Resources**

http://www.aha.org/advocacy-issues/emergreadiness/ebola/index.shtml

Communications to hospitals; Ebola preparedness calls; health communications; hospital resources; legal, regulatory, and ethical information; as well links to other resources

**United States Department of Labor Occupational Safety & Health Administration**

https://www.osha.gov/SLTC/ebola/additionalinformation.html

Resource links to general Ebola information; outbreak information; resources for workers and health-care workers; cleaning and decontamination information; information for airline cabin crews, mortuary workers, laboratory workers, emergency responders, and other types of workers; information on monitoring and surveillance and reporting tools

**Medscape Quiz: How Much Do You Really Know About Ebola Virus?**

http://reference.medscape.com/viewarticle/831792

**Medscape Ebola Virus Infection**

http://emedicine.medscape.com/article/216288-overview

Practice essentials, information on Ebola updates, signs/symptoms, diagnosis, management, and images

**Medscape Ebola Resource Center**

http://www.medscape.com/resource/ebola

Clinical guidelines, questions and answers, containment in hospitals, evaluation for patients; transmission information, news, and updates

## Online Articles

**Ebola: Clinician Concerns as Flu Season Begins**

http://www.medscape.com/viewarticle/833811

**Are Healthcare Providers Legally Obligated to Treat Ebola Patients?**

http://midlevelu.com/blog/are-healthcare-providers-legally-obligated-treat-ebola-patients

## Apps

**HealthMap: Outbreaks Near Me**

http://healthmap.org/formobile/

For the past 8 years, HealthMap, headed by a group at Boston Children’s Hospital, has provided real-time information on outbreaks of infectious diseases in an online format. This app is free and provides current surveillance, allowing tracking of emerging public-health threats. The website (healthmap.org) and mobile app Outbreaks Near Me provide users critical information on a wide range of emerging infectious diseases, including the Ebola virus.

**ImplementHIT**

implementhit.com/ebola/

Free Ebola training for physicians, advanced practitioners, and nurses via a mobile learning curriculum.
